# A Systematic Review of INGARCH Models for Integer-Valued Time Series

**DOI:** 10.3390/e25060922

**Published:** 2023-06-11

**Authors:** Mengya Liu, Fukang Zhu, Jianfeng Li, Chuning Sun

**Affiliations:** 1 School of Mathematics and Statistics, Central China Normal University, Wuhan 430079, China; 2 School of Mathematics, Jilin University, Changchun 130012, China; 3 School of Business, Zhengzhou University, Zhengzhou 450001, China

**Keywords:** INGARCH, count time series, conditional distribution, dynamic structure, robust estimation

## Abstract

Count time series are widely available in fields such as epidemiology, finance, meteorology, and sports, and thus there is a growing demand for both methodological and application-oriented research on such data. This paper reviews recent developments in integer-valued generalized autoregressive conditional heteroscedasticity (INGARCH) models over the past five years, focusing on data types including unbounded non-negative counts, bounded non-negative counts, Z-valued time series and multivariate counts. For each type of data, our review follows the three main lines of model innovation, methodological development, and expansion of application areas. We attempt to summarize the recent methodological developments of INGARCH models for each data type for the integration of the whole INGARCH modeling field and suggest some potential research topics.

## 1. Introduction

This paper reviews the development of modeling and inference for four types of integer time series, including the unbounded N-valued counts, Z-valued time series, bounded N-valued counts and multivariate counts. Firstly, the unbounded N-valued counts, also known as count time series, refer to discrete time series taking values in the range N={0,1,2,⋯}. For example, during an influenza outbreak, the number of new confirmed cases is reported daily, even down to each community. The analysis of such data is one of the fundamental tasks in epidemic forecasting and policy implementation (see Agosto and Giudici [1], Agosto et al. [2] and Giudici et al. [3] among others). Secondly, Z-valued count time series taking values in the range Z={⋯,−1,0,1⋯} are the appropriate tool to employ when attention is turned to, for example, the changes in athletic performance by the difference between the number of goals scored in each game and that in the previous one. Thirdly, the study of bounded count time series with a range of {0,⋯,n} for a given n∈N is also a concern, such as the rigorously recorded data set of water quality in the estuary. Finally, with the development of data acquisition technology and the expansion of storage space, multivariate count time series have emerged in various fields, and the related research is also developing.

For the above four types of integer time series, this review focuses on the the relevant research fields of the integer-valued generalized autoregressive conditional heteroscedasticity (INGARCH) models, which assume that the observations follow a discrete distribution and are conditioned on an accompanying intensity process that drives the dynamics, which are classified as observation-driven models. For instance, given the intensity process, the observations of a count time series follow a Poisson distribution and the intensity process is a linear combination of its lagged values and lagged observations (Fokianos et al. [4]). The diversity of count time series urges the INGARCH-type models to evolve to a broader domain. This is accompanied by the development of many subdivision directions, including the selection and even the creation of suitable conditional distributions, the exploration of nonlinear dynamic structures, the proof of stationarity and ergodicity, the relaxation of restrictions, the updating of methodologies, and so on. The purpose of this paper is to review recent developments, including the above subdivision directions, in INGARCH models over the past five years, as earlier work has been summarized. For the generally methodological development of count time series, not only INGARCH-type models, please refer to Weiß [5] and Davis et al. [6].

The INGARCH-type models for count time series are the most well-developed of these four types of integer-valued time series modeling, and they are reviewed in Section 2. On the one hand, to model some specific features of count time series, researchers have innovated the conditional distribution assumption. Indeed, Gorgi [7] and Qian and Zhu [8] focus on heavy-tailed count time series, while Silva and Souza [9] is more concerned with the generality of distribution and Souza et al. [10] further extends Silva and Souza [9] to the time-varying version. Accordingly, they all propose new conditional distribution assumptions that can model the features of interest. On the other hand, there are some new developments in the dynamic structure of INGARCH. For example, Weiß et al. [11] introduces the softplus function in the linear dynamic structure to implement the possibility of negative ACF; similar to Souza et al. [10], Roy [12] also focuses on the time-varying feature, but proceeds from the construction of a semi-parametric dynamic structure based on a Bayesian framework; some advances are made in the study of nonlinear dynamic structures with the help of threshold structures and the hysteresis model (Liu et al. [13], Chen and Khamthong [14], Liu et al. [15] and Chen et al. [16]). Moreover, when it comes to proving the existence and uniqueness of stationary distributions, which has been a challenging work in this field, the common approaches recently are based on approximation techniques, weak dependence or theoretical frameworks using the Feller property, e-chain and Lyapunov methods. In contrast, the absolute regularity for nonlinear GARCH and INGARCH models under a mild assumption are considered by Doukhan and Neumann [17], and a series of methodological studies are specifically reviewed in Section 2.

In contrast, the INGARCH-type models for the other three integer-valued time series are all still areas of ongoing research. The development patterns of all three areas are similar to that of the above-mentioned INGARCH-type models for count time series, including innovation in conditional distribution assumptions, flexibility in dynamic structure and methodological establishment, etc., but the challenges faced by each are different. For Z-valued time series, the difficulty of finding a suitable conditional distribution can be overcome, since it can be handled directly with the help of a sequence of binary or ternary random variables (Hu [18], Hu and Andrews [19] and Xu and Zhu [20]). However, the INGARCH-type models for Z-valued time series require more complex and subtle dynamic structures to implement, and the corresponding theoretical proofs are of higher difficulty. For bounded counts with range {0,⋯,n} for given n∈N, two research frameworks are currently divided into those based on vector form (Fokianos and Truquet [21]) and those based on scalar form (Weiß and Jahn [22], Chen et al. [23], Chen et al. [24] among others). The challenge encountered in the former is the computational stress of the estimation of matrix-type unknown parameters, while the difficulty in the latter lies in the rarity of suitable distributions, which are required to be discrete distributions with a range of values, and the accompanying proof of stationarity. Finally, the development of INGARCH-type models for multivariate numerical time series is still in its nascent stage. Lee et al. [25], Kim et al. [26] and Cui and Zhu [27] focus on the INGARCH-type models for bivariate count times series, but, subject to the development of multivariate Poisson distribution, more multivariate models related to these studies have not been extended. Another emerging area is the INGARCH-type models for time-varying network data (Armillotta and Fokianos [28], Armillotta et al. [29], Armillotta and Fokianos [30] and Tao et al. [31]), and many of the attempts that come with the challenge are worthwhile, such as optimization of conditional distributions, nonlinear dynamic structures and time-varying network data with upper bounds on the number of edges.

**Figure 1:**
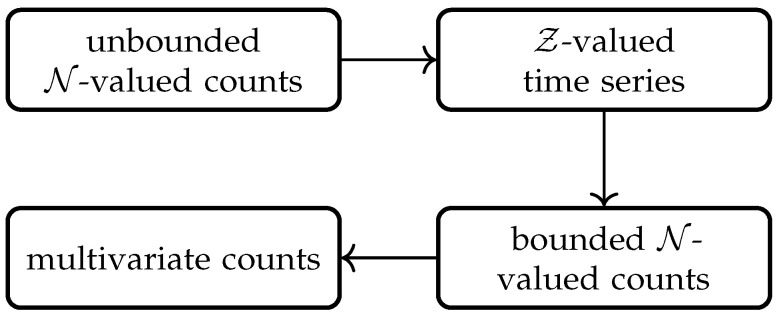
Flowchart of the types of time series reviewed.

The rest of the paper attempts to review the INGARCH-type models, which are presented below. Section 2, Section 3, Section 4 and Section 5 present the INGARCH-type models for unbounded N-valued counts, Z-valued time series, bounded N-valued counts and multivariate counts, respectively (Figure 1). Since there is much content related to unbounded N-valued counts, Section 2 is divided into three subsections to elaborate on the latest advances in models, methodologies and applications, but the other sections are also arranged in this way although they are not split again. Finally, we present some potential research topics from a personal perspective in Section 6.

## 2. Count Time Series

We consider count time series in this section, the possible outcomes of which are contained in N. Let ${\left\{X_{t}\right\}}_{t\in Z}$ be a count process, $x_{1},\cdots ,x_{T}$ be a finite set of observations, and ${Ϝ}_{t}$ be defined as σ-field generated by observations up to and including time *t*.

The INGARCH(p,q)-type models with p≥1 and q≥0 require two types of assumptions: first, a distributional assumption for $X_{t}$ conditioned on $X_{t-1},X_{t-2},\cdots$ is needed to guarantee the discrete range of $X_{t}$; secondly, the conditional mean $M_{t}=E\left(X_{t}|X_{t-1},\cdots \right)$ is required to be a linear or nonlinear expression in the last *p* observations and the last *q* conditional means, thus constructing a dynamic structure. For example, the Poisson INGARCH model assumes that $X_{t}$, conditioned on $X_{t-1},X_{t-2},\cdots ,$ is Poisson distributed with intensity parameters $M_{t}$ and

(1) $$M_{t}=\omega +\sum_{i=1}^{p}\alpha_{i}X_{t-i}+\sum_{j=1}^{q}\beta_{j}M_{t-j}.$$

This section begins with a review of recent modeling work on improving these two types of assumptions separately.

### 2.1. Recent Advances in Assumptions of Conditional Distribution

This subsection begins with the progress of research on count time series with outliers. For the INGARCH models, the common approach is to assume a heavy-tailed conditional distribution. Gorgi [7] introduced a heavy-tailed mixture of negative binomial distributions, known as the beta-negative binomial (BNB) distribution, as an distribution of $X_{t}$ conditioned on $X_{t-1},X_{t-2},\cdots$. See Table 1 for the specific definition of BNB. Relatedly, Qian and Zhu [8] was also concerned with heavy-tailed count time series and thus uses the generalized Conway–Maxwell–Poisson (GCOMP) distribution (in Table 1), which has one more parameter than the Conway–Maxwell–Poisson distribution, but provides a unified framework to handle over- or under-dispersed, zero-inflated, and heavy-tailed count data.

**Definition 1.**  *The following model is denoted by GCOMP-INGARCH(p,q),*

(2) $$X_{t}|{Ϝ}_{t-1}∼GCOMP\left(\lambda_{t},\nu ,r\right),\;\lambda_{t}=\alpha_{0}+\sum_{i=1}^{p}\alpha_{i}X_{t-i}+\sum_{j=1}^{q}\beta_{j}\lambda_{t-1},$$

*where $\alpha_{0}>0,\alpha_{i}\geq 0,\beta_{i}\geq 0,i=1,\cdots ,p,j=1,\cdots ,q,p\geq 1,q\geq 0,\nu >0$ and r<1. Specifically, the model (1) is GCOMP-INARCH(p) when q=0.*

According to the properties of the GCOMP distribution, the approximate conditional expectation and variance are given by

$$E\left(X_{t}|{Ϝ}_{t-1}\right)\approx \lambda_{t}+\frac{(2\nu -1)r}{2(1-r)},\;Var\left(X_{t}|{Ϝ}_{t-1}\right)\approx \frac{\lambda_{t}}{1-r},$$

and these are fundamentally the keys to the flexibility of the GCOMP-INGARCH(p,q) model. Qian and Zhu [8] also established some properties by assuming that model (2) is approximately stationary.

Additionally, Silva and Souza [9] proposes a general class of INGARCH models by introducing the mixed Poisson (MP) distribution proposed by Barreto-Souza and Simas [32]. This not only enriches the distribution types of INGARCH models, but also extends the negative binomial INGARCH process proposed by Zhu [33], and can even further evolves new models such as Poisson inverse Gaussian and Poisson generalized hyperbolic slope processes. Related works include Manaa and Bentarzi [34], Almohaimeed [35], Almohaimeed [36], and Cui and Wang [37], where Manaa and Bentarzi [34] focused on the expansion of time-varying parameters, which extends the time-invariant negative binomial INGARCH(1,1) studied by Zhu [33] to the periodic negative binomial INGARCH(1,1) model. Besides the flexibility of distributions, Silva and Souza [9] construct an expectation-maximization algorithm for estimating the parameters, in particular, the dispersion parameter. This provides a new framework for parameter estimation of the INGARCH-type models, which we believe also leaves a wide scope for further research.

**Definition 2**  (MP-INGARCH model)**.** *The process ${\left\{X_{t}\right\}}_{t\in Z}$ follows a MP-INGARCH(p,q) model if it satisfies*

(3) $$\left\{\begin{array}{c} X_{t}|{Ϝ}_{t-1}∼MP\left(\mu_{t},\phi \right),\;\;\forall t\in Z,\\ \mu_{t}=\alpha_{0}+\sum_{i=1}^{p}\alpha_{i}X_{t-i}+\sum_{j=1}^{q}\beta_{j}\mu_{t-j}, \end{array}\right.$$

*where ϕ is the dispersion parameter, $\alpha_{i}\geq 0,$ for i=0,1,⋯,p and $\beta_{j}\geq 0,$ for j=0,1,⋯,q, with p,q∈N.*

Note that the dispersion parameter ϕ in Definition 2 is independent of time *t*, and thus Souza et al. [10] is further extended for this setting. Indeed, the dispersion parameter is made time-varying, $\phi_{t}$, by creating a dynamic structure for it similar to that for the intensity parameter. The resulting model in Definition 3 is called a linear time-varying dispersion INGARCH (tv-DINGARCH) model (nonlinear tv-DINGARCH($p_{1}$,$p_{2}$, $q_{1}$, $q_{2}$) is omitted here). An interesting feature of the linear tv-DINGARCH processes is that to some extent it is more analogous to the original GARCH model than other INGARCH models. In particular, the mean of this model can be constant, while the variance depends on time as in an ordinary GARCH model. This feature is not possible to be accommodated by the standard INGARCH. Hence, they refer to the model as a pure INGARCH process to highlight the degree of association with GARCH that distinguishes it from other INGARCH models.

**Definition 3**  (Linear tv-DINGARCH model)**.** *A linear tv-DINGARCH(1,1,1,1) model $\left\{X_{t}\right\}$ is defined by $X_{t}|{Ϝ}_{t-1}∼MP\left(\mu_{t},\phi_{t}\right),\;\;\forall t\in Z,$ with*

(4) $$\lambda_{t}=\beta_{0}+\beta_{1}Y_{t-1}+\beta_{2}\lambda_{t-1},\;\;\phi_{t}=\alpha_{0}+\alpha_{1}Y_{t-1}+\alpha_{2}\phi_{t-1},$$

*where $\beta_{0},\alpha_{0}>0$ and $\beta_{1},\beta_{2},\alpha_{1},\alpha_{2}\geq 0$.*

In addition, zero- or zero-one-inflated INGARCH models are valuable in applications such as insurance. Lee and Kim [38] proposes more general multiple values-inflated INGARCH models in Definition 4. The conditional distribution q(·|η) containing only one unknown parameter is indeed a streamlined setup, but it cannot be ignored that the multiple inflated values will be accompanied by a simultaneous increase in the number of parameters $\rho_{m}$ and the resulting complexity can hardly be offset. From our personal point of view, a possible reason for such a dilemma is that the idea of a multi-inflated model constructed by superimposing indicator functions remains fundamentally indistinguishable from that of the zero-inflated model.

**Definition 4**  (multiple values-inflated INGARCH)**.** *Let$\left\{Y_{t},t\geq 1\right\}$ be a time series of counts taking values 0, 1, … following the multiple values-inflated INGARCH model with the conditional distribution of the one-parameter exponential family:*

(5) $$Y_{t}|{Ϝ}_{t-1}\;∼p\left(\cdot |\eta_{t}\right),\;\;X_{t}:=E\left(Y_{t}|{Ϝ}_{t-1}\right)=f_{\theta }\left(X_{t-1},Y_{t-1}\right),$$

*where ${Ϝ}_{t-1}$ is the σ-field generated by $\eta_{1},Y_{1},\cdots ,Y_{t},$ and $f_{\theta }\left(x,y\right)$ is a non-negative bivariate function defined on [0,∞)×N, depending on the parameter $\theta \in \Theta \subset R^{d}$, and p(·|·) is a probability mass function given by*

(6) $$\begin{array}{c} p\left(z|\eta \right)=\sum_{m=0}^{M-1}\left\{\rho_{m}+\left(1-\sum_{m=0}^{M-1}\rho_{m}\right)q\left(m|\eta \right)\right\}I\left(z=m\right)\\ +\left(1-\sum_{m=0}^{M-1}\rho_{m}\right)q\left(z|\eta \right)I\left(z\geq M\right),\\ q\left(z|\eta \right)=\exp\left\{\eta z-A\left(\eta \right)\right\}h\left(z\right),\;\;z\geq 0,\;\;and\;\;0\leq \rho_{m}<1. \end{array}$$

*Here, η is the natural parameter, A(·) and h(·) are known functions, and $B\left(\cdot \right):=A^{{}^{\prime }}\left(\cdot \right)$ exists and is strictly increasing. In Model (5), $Y_{t}|{Ϝ}_{t-1}∼p\left(\cdot |\eta_{t}\right)$ implicates $Y_{t}=F_{X_{t}}^{-1}\left(U_{t}\right),$ where $F_{x}\left(y\right)=\sum_{m=0}^{y}p\left(m|\eta \right)$ with $\eta =B^{-1}\left(x\right)$ and $U_{t}$ are i.i.d. uniform random variables over (0,1).*

An alternative approach to model generalization is to weaken the assumptions of the structural model allowing for more generalized link functions, and Wechsung and Neumann [39] make some contributions to the estimation of linkage functions. Wechsung and Neumann [39] considered a nonparametric version of the INGARCH(1,1) model, where the link function in the recursion for the variances was not specified by finite-dimensional parameters. This work completed the asymptotic analysis based on the mixed property, benefiting from the application of powerful exponential tail bounds in connection with a chaining argument. This work is highly theoretical, and shows in principle that a sufficiently regular link function of an INGARCH(1,1) process with hidden intensities can be estimated using a nonparametric least squares estimator, where the estimate is chosen from a truly nonparametric class of candidate functions. Further, the consistency rate of the estimator was shown to be nearly optimal. For practical purposes, Wechsung and Neumann [39] also reported an approximate version of the theoretical estimator.

The assumption about the distribution has also been approached from another perspective. Instead of specifying the conditional distribution, Aknouche and Demmouche [40] presents a double mixed Poisson autoregression whose conditional distribution is a superposition of two mixtures of Poisson distributions. It is more flexible compared to Poisson mixtures at the cost of just a few additional parameters. Further, Doukhan et al. [41] considered the mixture of nonlinear Poisson autoregressions, and Mao et al. [42] proposed a more general mixture INGARCH model, which includes s negative binomial mixture INGARCH of Diop et al. [43] and generalized Poisson mixture INGARCH models.

**Definition 5.**  *A generalized mixture INGARCH model is defined as follows:*

(7) $$\left\{\begin{array}{c} X_{t}=\sum_{k=1}^{K}1\left(\eta_{t}=k\right)Y_{kt},\\ E\left(Y_{kt}|{Ϝ}_{t-1}\right)=\lambda_{kt},\\ Var\left(Y_{kt}|{Ϝ}_{t-1}\right)=\nu_{k0}\lambda_{kt}+\nu_{k1}\lambda_{kt}^{2},\\ \lambda_{kt}=\alpha_{k0}+\sum_{i=1}^{p_{k}}\alpha_{ki}X_{t-i}+\sum_{j=1}^{q_{k}}\beta_{kj}\lambda_{k(t-j)}, \end{array}\right.$$

*where $\nu_{k0}\geq 0,\nu_{k1}\geq 0,$ but not simultaneously equal to zero, $\alpha_{k0}>0,\alpha_{ki}\geq 0,\beta_{kj}\geq 0$ for $i=1,\cdots ,p_{k},j=1,\cdots ,q_{k},$ 1(·) denotes the indicator function, ${Ϝ}_{t-1}$ indicates the information given up to time t−1, $\eta_{t}$ is a sequence of i.i.d. random variables with $P\left(\eta_{t}=k\right)=\alpha_{k},\;\alpha_{1}+\alpha_{2}+\cdots +\alpha_{K}=1,\alpha_{k}\geq 0$ and k=1,⋯,K. Furthermore, it is assumed that $X_{t-j}$ and $\eta_{t}$ are independent for all t and j>0, the variables $Y_{kt}$ and $\eta_{t}$ are conditionally independent given ${Ϝ}_{t-1}$ and $\alpha_{1}\geq \alpha_{2}\geq \cdots \geq \alpha_{K}$ for identifiability. If $\beta_{kj}=0$ for all $j=1,\cdots ,q_{k}$ and k=1,⋯,K, it reduces to the generalized mixture integer-valued ARCH model.*

### 2.2. Recent Advances in Dynamic Structures

This subsection reviews the latest advances in dynamic structures of INGARCH. Many recent works have made attempts in this area, but due to space limitations, we mainly focus on the essential dynamic structural innovations, some of which will not be mentioned one by one; for example, Souza et al. [10] also considered the inclusion of covariate/exogenous time series in tv-DINGARCH model, which enhances the applicability.

First, the INGARCH model with linear dynamic structure has been limited in its setting. Specifically, since the mean of a count random variable is a positive real number, the constraints $a_{0}>0$ and $a_{1},\cdots ,a_{p},b_{1},\cdots ,b_{q}\geq 0$ have to both hold in (1). This severely dampens the possibility of a negative ACF. The existing log-linear INGARCH model is an implementable solution to achieve negative ACF values, but at the cost of losing the linear conditional mean and the ARMA-like autocorrelation structure. Resolving the dilemma between the linear dynamic structure and a wider range of achievable ACF values is one of the key steps in the further development of the INGARCH-type models. The contribution of Weiß et al. [11] is to resolve this dilemma with the help of the softplus function. The definition of softplus INGARCH model is given as follows.

**Definition 6**  (softplus Poisson INGARCH model)**.** *$X_{t}$ follows from the softplus Poisson INGARCH model if satisfying*

(8) $$X_{t}|{Ϝ}_{t-1}:Poi\left(M_{t}\right),\;M_{t}=sp_{c}\left(\alpha_{0}+\sum_{i=1}^{p}\alpha_{i}X_{t-i}+\sum_{j=1}^{q}\beta_{j}M_{t-j}\right),$$

*where the softplus function $sp_{c}\left(x\right)=c\ln\left(1+\exp\left(x/c\right)\right)$ with c>0, and $\alpha_{0},\cdots ,\alpha_{p},\beta_{1},\cdots$, $\beta_{q}\in R$. The default choice for c is c=1.*

The softplus function $sp_{c}\left(x\right)$ avoids the drawback of not being differentiable in zero, while being approximately linear for x>0. The excellent properties of the softplus functions play a key role as they coincide with the breakthrough of the dilemma of INGARCH mentioned above.

What follows is a progression of time-varying features in INGARCH, which has been mentioned in the previous introduction of linear tv-DINGARCH processes in Definition 3, but Souza et al. [10] approached it from the distribution. Roy [12] proceeded from the construction of a semi-parametric dynamic structure, and proposed a time-varying autoregressive models for count time-series based on a Bayesian framework (Definition 7). This is the first attempt to model possibly autoregressive count time series with time-varying coefficients, and the success of this attempt can be attributed in part to the Bayesian framework.

**Definition 7**  (time-varying Bayesian INGARCH model)**.** *If the conditional distribution for $X_{t}$ given ${Ϝ}_{t-1}$ is $Poi(\lambda_{t})$ and*

(9) $$\lambda_{t}=\mu \left(t/T\right)+\sum_{i=1}^{p}a_{i}\left(t/T\right)X_{t-i}+\sum_{j=1}^{q}b_{j}\left(t/T\right)\lambda_{t-j},$$

*where the hierarchical prior on unknown functions μ(·), $a_{i}\left(\cdot \right)$ and $b_{i}\left(\cdot \right)$ are based on B-spline bases, $\left\{X_{t}\right\}$ is said to follow from the time-varying Bayesian INGARCH model.*

Take (1) with p=q=1 for example, the essential role of the original dynamic structure is to drive the time-varying nature of the conditional mean $M_{t}$, but where the parameters ω, $\alpha_{1}$ and $\beta_{1}$ do not vary with time. This means that the dynamic structure is allowed to take into account the heterogeneity changes at t−1 moments and the effects over the entire time period reflected by the time-constant parameters. In contrast, the semi-parametric time-varying dynamic structure (9) corresponds to further strengthening the time-varying property while weakening the average effect over the whole period. This modeling idea is better suited for rapidly changing count time series. In conclusion, Roy [12] contributes a new idea to the study of INGARCH from a Bayesian viewpoint. Its framework for the study of time-varying Bayesian INGARCH models is adapted to general non-stationary time series.

Next are some advances in the study of nonlinear dynamic structures with the help of threshold structures. The classical two-regime threshold autoregressive model allows for many properties associated with nonlinearity, and thus, both Liu et al. [13] and Chen and Khamthong [14] draw on this classical model to implement a nonlinear study of the dynamic structure of INGARCH, with the difference that the latter is based on the Markov switching approach and introduces covariates. It is worth mentioning that Lee and Hwang [44] proposed a generalized regime-switching INGARCH(1,1) model in a fashion similar to, yet different from, Chen and Khamthong [14], which also employs Markov chains with time-varying dependent transition probabilities. The difference is that Lee and Hwang [44] derives recursive formulas for the conditional probabilities of regimes in Markov chains given past information, starting from the Poisson parameters of the INGARCH(1,1) process.

**Definition 8.**  *The Markov switching INGARCHX model with state variables is defined by*

(10) $$p\left(X_{t}|{Ϝ}_{t-1}\right)=\left(\begin{array}{c} X_{t}+r-1\\ r-1 \end{array}\right){\left(\frac{1}{1+\lambda_{t}}\right)}^{r}\left(\frac{\lambda_{t}}{1+\lambda_{t}}\right),\;\;X_{t}\geq 0$$

(11) $$\lambda_{t}=\left\{\begin{array}{c} \alpha_{0}^{\left(1\right)}+\alpha_{1}^{\left(1\right)}X_{t-1}+\beta_{1}^{\left(1\right)}\lambda_{t-1},\;if\;s_{t}=1\\ \alpha_{0}^{\left(2\right)}+\alpha_{1}^{\left(2\right)}Y_{t-1}+\beta_{1}^{\left(2\right)}\lambda_{t-1},\;if\;s_{t}=2, \end{array}\right.$$

*where $s_{t}$ follows a first-order Markov chain with the following transition matrix:*

$$\vartheta =\left(\begin{array}{c} Pr\left(s_{t}=1|s_{t-1}=1\right)Pr\left(s_{t}=2|s_{t-1}=1\right)\\ Pr\left(s_{t}=1|s_{t-1}=2\right)Pr\left(s_{t}=2|s_{t-1}=2\right) \end{array}\right)=\left(\begin{array}{cc} p_{11} & p_{12}\\ p_{21} & p_{22} \end{array}\right)$$

*and $\alpha_{i}={\left(\alpha_{0}^{\left(i\right)},\alpha_{1}^{\left(i\right)},\beta_{1}^{\left(i\right)}\right)}^{\prime }$ is a non-negative parameter vector in state i. Naturally, changing the form of $\lambda_{t}$ yields the threshold INGARCHX as follows:*

$$\lambda_{t}=\left\{\begin{array}{c} \alpha_{0}^{\left(1\right)}+\alpha_{1}^{\left(1\right)}Y_{t-1}+\beta_{1}^{\left(1\right)}\lambda_{t-1},\;if\;Y_{t-d}\leq c\\ \alpha_{0}^{\left(2\right)}+\alpha_{1}^{\left(2\right)}Y_{t-1}+\beta_{1}^{\left(2\right)}\lambda_{t-1},\;if\;Y_{t-d}>c, \end{array}\right.$$

*where $Y_{t-d}$ is the threshold variable determining the dynamic switching mechanism of the model, d is a delay lag and c is the threshold value.*

The segmented dynamic structure may lead to sudden changes in the probability of the INGARCH process, which is a crux that needs to be improved. Li et al. [45] considered a hysteretic process with the hysteresis variable $\left\{S_{t}\right\}$ and the hysteresis zone $\left(r_{L},r_{U}\right]$, which makes the regime-switching mechanism more flexible. On this basis, Liu et al. [15] improved the self-excited threshold negative binomial autoregression (TNBAR) of Liu et al. [13] and proposed the self-excited hysteretic negative binomial autoregression (SEHNBAR) with the hysteresis variable $S_{t}=X_{t}$.

**Definition 9.**  *Let $\left\{N_{t},t\in Z\right\}$ be a sequence of the i.i.d. negative binomial process given in Liu et al. [13], then $\left\{X_{t}\right\}$ is said to follow the SEHNBAR model, if*

$$X_{t}=N\left(0,\lambda_{t}\right]$$

*with*

(12) $$\lambda_{t}=\left\{\begin{array}{cc} d_{1}+a_{1}\lambda_{t-1}+b_{1}X_{t-1}, & R_{t}=1,\\ d_{2}+a_{2}\lambda_{t-1}+b_{2}X_{t-1}, & R_{t}=0, \end{array}\right.$$

*where $d_{i},a_{i},b_{i}>0,i=1,2$,*

(13) $$R_{t}=\left\{\begin{array}{cc} 1, & X_{t-1}\leq r_{L},\\ 0, & X_{t-1}>r_{U},\\ R_{t-1}, & otherwise. \end{array}\right.$$

*$R_{t}$ is the regime indicator with the hysteresis variable $Y_{t-1}$, and $\left(r_{L},r_{U}\right)$ are boundary parameters of the hysteresis zone satisfying $0\leq r_{L}\leq r_{U}<\infty$.*

$\lambda_{t}$ is at the lower regime when $X_{t-1}<r_{L}$, while at the upper regime when $X_{t-1}>r_{U}$. If $X_{t-1}$ falls within the hysteresis zone, the regime indicator remains unchanged, which means that the regime indicator at time *t* is the same with that at t−1. Formally, the hysteresis model with piecewise linear structure enjoys a more flexible regime-switching mechanism. Another less intuitive but proven advantage is that the lagged observations on which $\lambda_{t}$ relies are infinitely far away, which is the key difference between hysteresis models and traditional threshold models. Chen et al. [16] also proposed a similar INGARCH model based on the Bayesian framework.

Aknouche and Scotto [46] proposes a multiplicative INGARCH model, which is defined as the product of a unit-mean integer-valued i.i.d. sequence, and an integer-valued dependent process defined as a binomial thinning operation of its own past and of the past of the observed process. This model combines some features of the INGARCH, the autoregressive conditional duration, and the integer autoregression processes, so it can be used to model high overdispersion, persistence, and heavy-tailedness. Furthermore, Weiß and Zhu [47] propose an integer-valued analog of multiplicative error models based on a multiplicative operator, and the resulting models are closely related to the class of INGARCH models.

Last but not least, some migratory research works that have received attention in other fields but are poorly known in the INGARCH model are also of interest. Sim et al. [48] established the overall framework for the study of general-order INGARCH(p,q) models without the restriction p=q=1. Similarly, the purpose of Tsamtsakiri and Karlis [49] was to select the most appropriate order of INGARCH(p,q) using a trans-dimensional Bayesian approach, and Tian et al. [50] focused on order shrinkage and selection for the INGARCH(p,q) model. Furthermore, the temporal aggregation and systematic sampling, which were widely studied in continuous-valued time series, have received the attention of Su and Zhu [51] in integer-valued time series.

**Figure 2:**
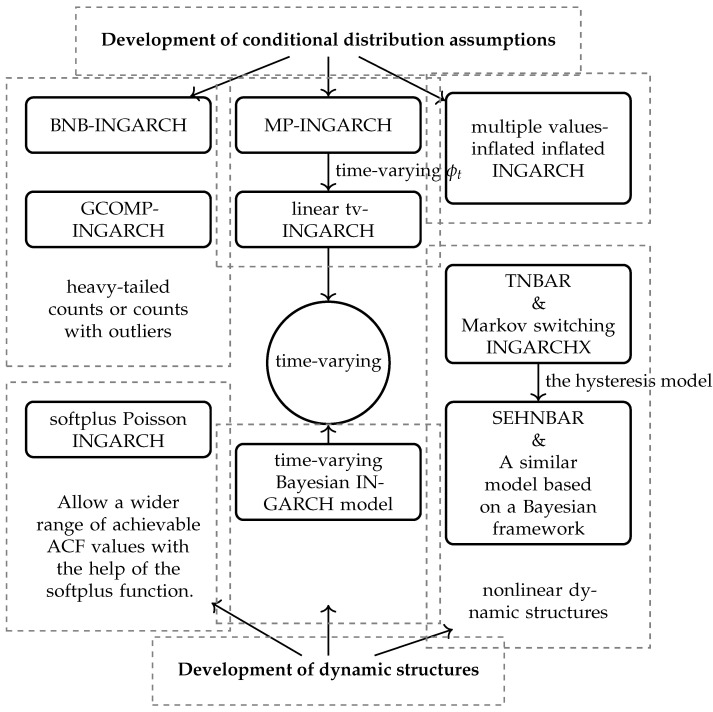
Flowchart of some major INGARCH-type models for unbounded counts.

For clarity, we have sorted out the main relationships of the models reviewed in this section in Figure 2.

### 2.3. Methodologies

There are many theory-oriented advances. The proofs for the existence and uniqueness of stationary distributions in the above-mentioned literature or in the earlier INGARCH literature are based on approximation techniques, the weak dependence or the theoretical framework using the Feller property, e-chain and Lyapunov’s method. In contrast, to prove the existence and uniqueness of a stationary distribution and absolute regularity for nonlinear GARCH and INGARCH models under a mild assumption, Doukhan and Neumann [17] treated $Z_{t}=\left(X_{t},\cdots ,X_{t-p+1},\lambda_{t},\cdots ,\lambda_{t-q+1}\right)$ as a time-homogeneous Markov chain where $\left\{\lambda_{t}\right\}$ is the accompanying process of random intensities, and compensated for missing Feller properties with coupling results. Specially, besides a geometric drift condition, only a semi-contractive condition was imposed, which means a subgeometric, rather than the more usual geometric, decay rate of the mixing coefficients. This result not only enriches the theoretical proof technique, but also broadens the application area of the INGARCH model.

Further, supposed that $\left\{X_{t}\right\}$ follow from Poisson INGARCH, Neumann [52] use the contraction property twice: first, under the contraction condition of the intensity process, Neumann [52] obtained the contraction property of the Markov kernel connected with $Z_{t}$ in terms of a suitable Wasserstein metric, and then the existence and uniqueness of a stationary distribution follows via the Banach fixed point theorem; next, the almost effortlessly absolute regularity of the count process was established by using the contraction property once more; finally, Neumann [52] constructed a coupling of the original and the bootstrap process, and proved the existence and uniqueness of a stationary version of this joint process as well as absolute regularity of the joint count processes. Notably, the last item implies that the model-based bootstrap method proposed by Neumann [52] is more general than most of the existing papers on the consistency of bootstrap. Specifically, the bootstrap process mimics the random behavior of the original counting process, rather than being limited to the plausibility of certain specific statistics. More broadly, Doukhan et al. [53] derived the absolute regularity at a geometric rate not only for stationary Poisson GARCH processes, but also for models with an explosive trend. Recently, Aknouche and Francq [54] considered the existence of a stationary and ergodic solution of a general Markov-Switching autoregressive conditional mean model, of which the INGARCH model is one of the variants.

The contribution of Douc et al. [55] is establishing the necessary and sufficient conditions for the identifiability of observation-driven models including the pure INGARCH model and its numerous extensions, such as the pure INGARCH model with thresholds or exogenous covariates.

Recent advances in estimation methods regarding the INGARCH model are reviewed as follows. One of the main reasons for the utility of negative binomial models is that Poisson INGARCH is less flexible than models based on overdispersed conditional distributions when modeling overdispersed series. However, parameter estimation for a negative binomial INGARCH model is usually implemented based on Poisson quasi-maximum likelihood estimation (quasi-MLE or QMLE), where the pitfall is that Poisson-QMLE is likely to fail to achieve its full asymptotic efficiency with the presence of overdispersion. To clear this hurdle, Aknouche et al. [56] proposed two negative binomial QMLEs (NB-QMLEs), including the profile NB-QMLE calculated while arbitrarily fixing the dispersion parameter of the negative binomial likelihood, and the two-stage NB-QMLE consisting estimation for both conditional mean and dispersion parameters. Similarly, the two-stage weighted least square estimators (WLSEs) proposed by Aknouche and Francq [57] are general for time series data, and feasible for the INGARCH model. WLSEs can be implemented without fully specifying the conditional distribution or time series structure, and enjoy the same consistency properties as QMLEs when the conditional distribution is mis-specified, even if the conditional variance is mis-specified. Additionally, a data-driven strategy was identified to find asymptotically optimal WLSEs. This actually provides prerequisite support for further relaxation of the conditional distribution assumption for the count time series. For the model in Aknouche and Scotto [46], parameter estimation is conducted by using a two-stage WLSE, and Xu et al. [58] considered a saddlepoint MLE for a special case of this model.

The commonly used MLE method is highly influenced by outliers, so there are several works dedicated to establish robust estimation methods. For Poisson INGARCH models, Li et al. [59] proposed a robust M-estimator by using a new loss function inspired by the Tukey’s biweight function. It is the construction of this loss function that contributes to this work. One of the disadvantages of Tukey’s function is that it drops to zero so that the effect of very large outliers or leverage points is completely suppressed, which implies the possibility of multiple solutions to the estimated equations. Then, an intuitive idea is to construct a hybrid loss function that does not fully suppress the effects of very large outliers or leverage points. Li et al. [59] proposed a new loss function by twofold improvement. Along similar lines, Xiong and Zhu [60] introduced a robust estimation for the one-parameter exponential family INGARCH(1,1) models.

Moreover, Kim and Lee [61] used the minimum density power dispersion estimator as a robust estimator for INGARCH models whose conditional distribution belongs to the one-parameter exponential family. There are two advantages to this approach: the first is simplicity, i.e., it contains only a single tuning parameter that controls the trade-off between robustness and efficiency; the second is the ability to balance robustness and efficiency, providing considerable robustness while retaining high levels of efficiency as the tuning parameter approaches zero. Further, Xiong and Zhu [62] and Xiong and Zhu [63] used the Mallows’ quasi-likelihood estimator and the minimum density power dispersion estimator as a robust estimator for negative binomial INGARCH models, respectively. For negative binomial INGARCH models, Elsaied and Fried [64] also developed several robust estimators including robustifications of method of moments and ML-estimation, one of which was an alternative to the robust estimator proposed by Xiong and Zhu [63].

Another drawback of MLE for INGARCH models is that numerical results are sensitive to the choice of initial values. Hence, Li and Zhu [65] proposed the mean targeting estimation, which is an analogue to variance targeting estimation used in the GARCH model. In addition, there have been some other advances in estimation. Jo and Lee [66] introduced the mean targeting QMLE based on INGARCH models, which provides a new perspective. When shifting to focus on more specific estimation problems, it is a common problem that the estimation performance of the intercept parameter is inferior to that of other parameters, either in the Poisson or negative binomial INGARCH model. Integrating the likelihood function by assuming a conditional distribution is one option to eliminate this obstacle. Hence, Pei and Zhu [67] adopted the marginal likelihood to estimate the intercept parameter in the negative binomial INGARCH model.

There are also some areas that are of interest to scholars. For example, to test the parameter variation of INGARCH, the cumulative sum (CUSUM) statistics has been the most popular method in recent years (Lee and Lee [68], Lee et al. [69], Lee [70], Vanli et al. [71], Weiß and Testik [72]), and Lee and Kim [73] reviewed a recent progress regarding the change point test for integer-valued time series models. Further, as with the techniques employed to present robust estimates, Kim and Lee [74] introduced a robust change point test based on density power divergence. Michel [75] considered the limiting distribution of the INGARCH(1,1) with α+β=1.

### 2.4. Applications

The practical application of INGARCH models has developed considerably in recent years, especially in the period of COVID-19 when there is a strong demand for analysis of count time series data such as daily new infections in various countries or regions. This has also given rise to many valuable research topics. Agosto and Giudici [1] focused on COVID-19 contagion and digital finance, and presented Poisson INGARCH of the daily newly observed cases to understand the contagion dynamics of the COVID-19. In addition, the purpose of Agosto et al. [2] is to monitor COVID-19 contagion growth and came to the interesting conclusion that policy measures aimed at reducing infection are very useful when the it is at its peak and can reduce reproduction rates. Souza et al. [10] considered the tv-DINGARCH model with covariate/exogenous time series and applied to the daily number of deaths due to COVID-19 in Ireland. In contrast, Roy [12] focused specifically on the data for New York City because the epidemic status in this city lasted for a month and with the help of the ongoing blockade, it recovered significantly within about three months. Hence, it is this temporal variability in the data that drew Roy [12] to explore its trends by the time-varying Bayesian INGARCH model. Similarly, Giudici et al. [3] was also concerned about the time-varying features of COVID-19 and proposed Bayesian time-dependent Poisson autoregressive models. Additionally, Gning et al. [76] focused on COVID-19 in Senegal and China. Moreover, the dynamics of COVID-19 infectivity in Saudi Arabia were evaluated in Alzahrani [77] by using two statistical models, namely the log-linear Poisson autoregressive model and the ARIMA model. The results of this study showed that the log-linear Poisson autoregressive model had superior predictive performance. At the same time, many application-oriented works have actually proposed new models to meet the requirements. For example, Xu et al. [78] proposes a comprehensive adaptive log-linear zero-inflated generalized Poisson INGARCH to describe crime counts in Byron and Australia, and the features of this data set include autocorrelation, heteroscedasticity, overdispersion and excessive number of zero observations.

In addition to COVID-19, INGARCH-type models are employed in other areas such as stock trading, co-tracking of commodity marketsand so on (Chen and Khamthong [79], Agosto and Raffinetti [80], Jamaludin et al. [81], Algieri and Leccadito [82], Aknouche et al. [83], Berentsen et al. [84], Cerqueti et al. [85]). It is worth mentioning that the INGARCH-type model has been favored for human influenza research even before the outbreak of COVID-19. Specifically, Chen et al. [86] involves the INGARCH-type model when examining the causal relationship between environmental fine particulate matter and human influenza in Taiwan. The study on traffic forecasting of Kim [87] affirms the value of the INGARCH model for applications. The prediction model of Kim [87] was generated by estimating the parameters of the INGARCH process and predicting the Poisson parameters of the future step ahead process using conditional MLE methods and prediction procedures, respectively. They came to the conclusion: “INGARCH captures the characteristics of network traffic better than other statistical models, it is more tractable than neural networks (NN), overcomes the black-box nature of NN, and some statistical models perform comparable or even better than NN, especially when there is insufficient data to apply deep NN”. Another application that tends to be humanistic and social Anavatan and Kayacan [88], the aim of which was to reveal the relationship between the number of femicide, female unemployment rate, male unemployment rate and the amount of information in Turkey by using INGARCH model.

In summary, the application scenarios of the INGARCH-type models can be as specific as a vehicle prediction at an intersection or as macro as a humanistic exploration of a country, and what is more valuable is that the exploration about their application still remains expected and promising.

## 3. Z-Valued Time Series

The previous section is concerned with count time series (i.e., N-valued time series). However, time series that allow both non-negative and negative integer values (i.e., Z-valued time series) are also worth investigating, whose research value is reflected in the following aspects: first, there are many typical application areas for Z-valued time series, e.g., describing score gaps in sports, trading changes in finance (Xu and Zhu [20]); secondly, the non-stationary property embodied in such time series is also of interest, such as differenced series that are initially non-stationary (Gonçalves and Mendes-Lopes [89]). Let ${\left\{Z_{t}\right\}}_{t\in Z}$ be a Z-valued process, and $z_{1},\cdots ,z_{T}$ be a finite set of observations. A recent study on the mixing properties of Z-valued GARCH processes can be found in Doukhan et al. [90]. Below, we review contributions on Z-valued time series in recent years.

Firstly, the Skellam distribution is introduced into the INGARCH-type models as a conditional distribution. Alomani et al. [91] proposed the Skellam GARCH(1, 1) model defined in Definition 10, where the Skellam is the distribution of the difference of two independent Poisson variates and thus allows for both non-negative and negative integer-valued variables. The specific definition of the Skellam distribution is placed in Table 2. The benefits of the Skellam INGARCH model are that they allow time-varying variance and nonstationarity in the mean for time series, and the conditional maximum likelihood and conditional least squares methods have been developed for estimation of the parameters.

**Definition 10.**  *$\left\{Z_{t}|{Ϝ}_{t-1}\right\}$ follow symmetric Skellam $\left(\frac{\sigma_{t|t-1}^{2}}{2},\frac{\sigma_{t|t-1}^{2}}{2}\right)$ with the conditional variance satisfying*

(14) $$\sigma_{t|t-1}^{2}=\omega +\alpha Z_{t-1}^{2}+\beta \sigma_{t-1|t-2}^{2},\;\;t\geq 2,$$

*In (14), the parameters ω, α and β satisfy the following constraints: ω>0, 0<α<1, 0<β<1 and α+β<1, which are necessary and sufficient for stationarity of the process (14). We refer to the above model as Skellam GARCH(1, 1).*

The drawback of the symmetric Skellam INGARCH(1,1) in Definition 10 is that only the simplest case is considered, i.e., the conditional expectation value is equal to zero. Cui et al. [92] proposes an asymmetric Skellam INGARCH to eliminate this limitation. Furthermore, the modified Skellam (MS) distribution (see Table 2) is introduced and thus the modified Skellam INGARCH in Definition 11. Specifically, Cui et al. [92] added a new parameter, γ, to the standard Skellam distribution whose mission is to control the distance between the probabilities $P(Z_{t}=0)$ and $\min\{P\left(Z_{t}=1\right),P\left(Z_{t}=-1\right)\}$ by its magnitude and sign. Hence, the modified Skellam INGARCH can flexibly compensate for the over- or under-representation of specific integers (−1,0,1).

**Definition 11**  (Modified Skellam INGARCH model)**.** *Consider the following model:*

$$\begin{array}{cc} Z_{t}|{Ϝ}_{t-1} & ∼MS(\gamma ,\lambda_{t}^{2},\lambda_{t}^{*2}),\\ \lambda_{t}^{2} & =\alpha_{0}+\alpha_{1}Z_{t-1}^{2}+\beta_{1}\lambda_{t-1}^{2},\\ \lambda_{t}^{*2} & =\alpha_{0}^{*}+\alpha_{1}Z_{t-1}^{2}+\beta_{1}\lambda_{t-1}^{*2}, \end{array}$$

where $\gamma \in (\max_{t}\left\{-P_{0t}/\Delta_{t}\right\},\min_{t}\{2\min(P_{-1t}$, $P_{1t})/\Delta_{t}\})$, $\Delta_{t}=P_{0t}-\min\left(P_{-1t},P_{1t}\right)>0$, $P_{qt}=P_{MS}\left(X_{t}=q\right)$, q∈Z, $\alpha_{0}>0$, $\alpha_{0}^{*}>0$, $\alpha_{1}\geq 0$, $\beta_{1}\geq 0$. The above model is denoted by MS-INGARCH(1, 1). Note that for γ=0, we recover the AS-INGARCH(1, 1) model.

Another path to modeling the Z-valued time series is to extend the N-valued INGARCH models by introducing a sequence of i.i.d. binary random variables independent of $\left\{Z_{t}\right\}$, $\left\{Q_{t}\right\}$, taking values at 1 and −1 with equal probability 0.5, such as a two-sided Poisson distribution. For example, Hu [18] and Hu and Andrews [19] proposed a Poisson Z-valued Glosten–Jagannathan–Runkle GARCH (PZG) model as follows.

**Definition 12.**  *We call $\left\{Z_{t}\right\}$ an integer-valued asymmetric GARCH process of orders p and q, if for all t∈Z,*

$$\begin{array}{cc} Z_{t} & =Q_{t}X_{t},\\ X_{t}|{Ϝ}_{t-1} & ∼Pois(\lambda_{t}),\\ \lambda_{t} & =\left(\sqrt{1+4\eta_{t}}-1\right)/2,\\ \eta_{t} & =\alpha_{0}+\sum_{i=1}^{p}\alpha_{i}\left(\right|Z_{t-i}{\left|-\gamma Z_{t-i}\right)}^{2}+\sum_{j=1}^{q}\beta_{j}\eta_{t-j}. \end{array}$$

*Hence, $\left\{X_{t},t\in Z\right\}$ is a non-negative integer-valued stochastic process; conditioned on past information up to and including time t−1, $X_{t}$ has Poisson distribution with mean $\lambda_{t}$. So that $\left\{\eta_{t}\right\}$ and $\left\{\lambda_{t}\right\}$ are positive, we assume parameter $\alpha_{0}>0$ and parameters $\alpha_{i},\beta_{j}$, for i=1,⋯,p, j=1,⋯,q, are all non-negative, with model orders p≥1,q≥0.*

It can be seen that a two-sided Poisson distribution is employed and the structure of $\eta_{t}$ is inspired by the Glosten–Jagannathan–Runkle GARCH (GJR-GARCH, Glosten et al. [93]). The main role of GJR-GARCH here is to portray asymmetric responses in the volatility of Z-valued time series, such as the presence of leverage effects in financial time series, and is thus the highlight of the PZG model. Furthermore, Xu and Zhu [20] proposed the geometric Z-valued GJR-GARCH model based on the shifted geometric distribution that is more flexible than Poisson distribution. Additionally, Xu and Zhu [20] expanded from a two-side equality probability of 0.5 to a broader form where the ratio of positive, negative and zero values can be controlled by a parameter ρ (Definition 13).

**Definition 13.**  *The geometric Z-valued GJR-GARCH model is defined as*

$$\begin{array}{cc} Z_{t} & =Q_{t}^{*}X_{t},\\ X_{t}|{Ϝ}_{t-1} & ∼SGe(p_{t}),\\ p_{t} & =\frac{\sqrt{\rho^{2}+4\rho \lambda_{t}}-\rho }{\lambda_{t}},\\ \lambda_{t} & =\omega +\sum_{i=1}^{p}\alpha_{i}\left(\right|Z_{t-i}{\left|-\gamma_{i}Z_{t-i}\right)}^{2}+\sum_{j=1}^{q}\beta_{j}\lambda_{t-j}, \end{array}$$

where SGe(·) denotes the shifted geometric distribution, ω>0,$|\gamma_{i}|\leq 1$, $\alpha_{i}\geq 0$, $\beta_{j}\geq 0$, for i=1,⋯,p, j=1,⋯,q, p≥1, q≥0. Specially, $\left\{Q_{t}^{*}\right\}$ is a sequence of i.i.d. random variables taking values at 1, 0 *and −1 with probabilities ρ, 1−2ρ and ρ, respectively, where $0<\rho <\frac{1}{2}$.*

## 4. Bounded Count Time Series

In what follows, ${\left\{B_{t}\right\}}_{t\in Z}$ consists of bounded counts with range {0,⋯,n} for given n∈N. This type of data also includes categorical time series, with binary time series being a special case. In terms of the INCARCH-type models, the study of $\left\{B_{t}\right\}$ differs from that of unbounded counts $\left\{X_{t}\right\}$ in two ways: one is that the candidates for the conditional distribution are required to be discrete distributions with bounded range of values; the other is that the dynamic structure of the conditional mean needs to be adjusted accordingly to the constraints of parameters of the conditional distribution, which leads to the possibility that the dynamic structure cannot be directly attached to the conditional mean, but some kind of functional transformation of the conditional mean. Accordingly, researchers have been bursting at the seams with recent innovative work in these two areas.

The definition of the bound INGARCH (BINGARCH) model is obtained by the distributional assumption that $B_{t}$ is generated by a bounded-count distribution and the normalized conditional mean $P_{t}=\frac{1}{n}E\left(X_{t}|X_{t-1},\cdots \right)$, where

(15) $$P_{t}=a_{0}+\sum_{i=1}^{p}a_{i}X_{t-i}/n+\sum_{j=1}^{q}b_{j}P_{t-j}$$

with the additional constraint $a_{0}+\sum_{i=1}^{p}a_{i}+\sum_{j=1}^{q}b_{j}<1$.

First, for convenience, we assume that $B_{t}$ is generated by the conditional binomial distribution $Bin(n,P_{t})$.

(16) $$B_{t}|{Ϝ}_{t-1}∼Bin\left(n,P_{t}\right)$$

Then, as with the constraints on the INGARCH-type model with linear dynamic structure mentioned previously, the BINGARCH-type model also requires constraints $a_{0}>0$ and $a_{1},\cdots ,a_{p},b_{1},\cdots ,b_{q}\geq 0$ to ensure positive $P_{t}$ in (15). In line with the idea of Weiß et al. [11], Weiß and Jahn [22] solved this puzzle in the BINGARCH-type model with the help of the soft-clipping functions, which enables the migration and application of this framework proposed by Weiß et al. [11]. Specially, Weiß and Jahn [22] considered the normalized conditional mean as follows:

(17) $$P_{t}=f\left(\alpha_{0}+\sum_{i=1}^{p}\alpha_{i}X_{t-i}/n+\sum_{j=1}^{q}\beta_{j}P_{t-j}\right).$$

where f(·) was set to be the soft-clipping function

(18) $$f\left(x\right)=sc_{\frac{c}{n}}\left(x\right)=\frac{c}{n}\ln\left(\frac{1+\exp(\frac{nx}{c})}{1+\exp\left(\frac{n(x-1)}{c}\right)}\right).$$

**Definition 14.**  *$\left\{B_{t}\right\}$ is said to follow the soft-clipping binomial INGARCH model if satisfying (16), (17) and (18). The model would be well-defined without any further restrictions, but some reasonable constraints such as $|\alpha_{i}\left|,\right|\beta_{j}|<1$ for i=1,⋯,p and j=1,⋯,q, and $\alpha_{0}\in \left(0,1+p+q\right)$ are needed.*

The soft-clipping binomial INGARCH was employed in Weiß and Testik [72] once more. The fascinating question considered by Weiß and Testik [72] is how the performance of the control chart is affected if the CUSUM control chart is designed based on the assumption of a completely linear data generation process, while the true one is only approximately linear. Weiß and Testik [72] aptly exploits the fact that the soft-clipping binomial INGARCH is an approximately linear counterpart of BINGARCH, and draw the conclusion that, in general, chart designs are robust to model mis-specification when parameters are specified, whereas the opposite result is obtained when the parameters are estimated.

The binomial distribution is a traditional choice for studying bounded count time series, as in (16), due to its simple form and relatively well-established properties. The motivation for the innovation against the conditional distribution is that there is a fixed relationship between the variance and the mean of the binomial distribution, denoted as the binomial index of dispersion (BID), similar to the equidispersion property of Poisson. The BID for a random variable *X* taking values in N is defined as

$$\mathrm{BID}=\frac{n\mathrm{Var}(X)}{E(X)(n-E(X))}.$$

Additionally, the BID of the binomial random variable is calculated to be 1, which indicates that (16) is not competent for modeling data with BID >1. Hence, Chen et al. [23] proposed a new class of INGARCH models with beta-binomial (BB) variation, which is a generalization of Chen et al. [94], in Definition 15, where the BID of BB distribution takes values in the interval (1,n). The specific form of the beta-binomial distribution is available in Table 3. To analyze the high volatility in time series counts, covariates were further introduced by Chen et al. [24], and thus a covariate-driven beta-binomial INGARCH model was proposed in Definition 16.

**Definition 15.**  *Let $\theta ={\left(\theta_{1},\cdots ,\theta_{d}\right)}^{⊤}$ be the vector of parameters. Then, the beta-binomial GARCH(1,1) model is defined as:*

(19) $$B_{t}|{Ϝ}_{t-1}:BB\left(n,p_{t},\phi \right),\;Y_{t}:=np_{t}:=g_{\theta }\left(Y_{t-1},B_{t-1}\right),t=1,2,\cdots ,$$

*where $g_{\theta }\left(Y_{t-1},B_{t-1}\right)$ is a non-negative and continuous function in terms of each $\theta_{j}$ for a given $Z_{t-1}$ and $Y_{t-1},\forall j=1,2,\cdots ,d.$*

**Definition 16.**  *Let $C_{t}=\left(C_{1t},C_{2t},\cdots ,C_{dt}\right)$ be a d-dimensional exogenous covariate vector. Then, the logit-BBGARCHX(1,1) model is defined as:*

(20) $$B_{t}|{Ϝ}_{t-1}:BB\left(n,P_{t},\phi \right),logit\left(p_{t}\right)=\omega +\alpha logit\left(p_{t-1}\right)+\beta B_{t-1}+f\left(C_{t-1},\gamma \right),\;t\in Z,$$

*where $logit\left(x\right)=\log\left(x/\left(1-x\right)\right),\forall x\in \left(0,1\right),f\left(\cdot ,\gamma \right):R^{d}\to R,\left(\omega ,\alpha ,\beta ,\phi \right)$ is the parameter vector with ω∈R,ϕ∈(0,1), |β|<4(1−|α|) and |α|<1, and γ is the additional parameter vector involving in f.*

The beta-binomial distribution employed in Chen et al. [23] and Chen et al. [24] allow to model bounded data with under-dispersion. Then, Chen [95] turned its attention to a rare case, i.e., an under-diversified pseudo-binomial data set. It is the discrete beta (DB) distribution that competently models bounded data with under-dispersion, equiv-dispersion, and over-dispersion. Motivated by this and the soft-clipping function used in Weiß et al. [11], Chen [95] proposed a new soft-clipping discrete beta GARCH model as follows.

**Definition 17.**  *The soft-clipping discrete beta GARCH(1,1) model is defined by*

(21) $$\left\{\begin{array}{c} B_{t}|{Ϝ}_{t-1}∼DB\left(n_{bot},n_{top},p_{t},\tau \right),\\ p_{t}=sc_{c}\left(\omega +\alpha_{1}p_{t-1}+\beta_{1}B_{t-1}/n_{top}\right), \end{array}\right.$$

*where the definition of DB is placed in Table 3, $sc_{c}\left(\cdot \right)$ is defined in (18), $|\alpha_{1}\left|+\right|\beta_{1}|\;<1$, $n_{bot}=0$ or 1 and $n_{top}\in N$ is the predetermined upper limit of the range.*

Categorical time series are also a type of bounded count-valued time series. In the study of INGARCH-type models, categorical time series are usually presented in vector form, which can be modeled by an autoregressive multinomial logistic time series model with a latent process and is defined by a GARCH-type recursive equation. Suppose that we observe a process with state space {0,1,⋯,n} and define a (n−1)-dimensional vector $Y_{t}={\left(Y_{1t},Y_{2t},\cdots ,Y_{(n-1)t}\right)}^{⊤}$, for 1≤t≤n, such that

(22) $$Y_{kt}=\left\{\begin{array}{c} 1,\;\mathrm{if}\;\mathrm{the}\;k\mathrm{th}\;\mathrm{category}\;\mathrm{is}\;\mathrm{observed}\;\mathrm{at}\;\mathrm{time}\;t,\\ 0,\;\mathrm{otherwise}, \end{array}\right.$$

for all k=1,2,⋯,n−1. Moreover,

$$p_{kt}=P\left(Y_{kt}=1|{Ϝ}_{t-1}\right),\;\;1\leq j\leq N-1,$$

is defined as a vector of conditional probabilities and $p_{t}\equiv {\left(p_{1t},p_{2t},\cdots ,p_{(n-1)t}\right)}^{\prime }$. For the last category *n*, set $Y_{nt}=1-\sum_{k=1}^{n-1}Y_{kt}$ and $p_{nt}=1-\sum_{k=1}^{n-1}p_{kt}$. The dynamic structure is dependent on $p_{t}$. For instance, the following linear dynamic structure is a classic:

$$p_{t}=d+Ap_{t-1}+BY_{t-1},$$

where *d* is a vector and *A*, *B* are matrices of appropriate dimensions. It is easy to see that the disadvantage of this modeling approach in application is the problem of using multidimensional methods to deal with univariate data resulting in increased pressure on parameter estimation and other troubles. However, its theoretical development is being gradually refined. Fokianos and Truquet [21] employed a useful coupling technique to study the ergodicity of infinite-order finite-state stochastic processes, making significant improvements to previous conditions on the stationarity and ergodicity of these models.

In addition to dealing with categorical time series in vector form, Liu et al. [96] proposed a simple and less computationally stressful way of modeling, which was essentially an innovative approach to conditional distributions from an application perspective. Liu et al. [96] first introduced a new zero-one-inflated bounded Poisson (ZOBP) distribution defined as

(23) $$P\left(B=k\right)=p_{1}I_{\left\{k=0\right\}}+p_{2}I_{\left\{k=1\right\}}+\left(1-p_{1}-p_{2}\right)\frac{\lambda^{k}/k!}{\sum_{i=0}^{M}\lambda^{i}/i!},\;\;k=0,1,\cdots ,M,$$

where $p_{1}\geq 0$ and $p_{2}\geq 0$ are the inflated parameters for the states 0 and 1, respectively, with the constraint $p_{1}+p_{2}<1$, λ>0 is the intensity parameter and the integer M≥2 is a given upper bound. This distribution is suitable for depicting data for air quality classes that are predominantly excellent, where 0 and 1 represent excellent and good air quality, respectively. Liu et al. [96] defined a new INGARCH-type model based on the ZOBP distribution. For ease of presentation, we denote the ZOBP distribution in (23) with $\left(p_{1},p_{2}\right)=\left(0,0\right)$ by $P^{*}\left(\lambda ,M\right)$. Let $\left\{D_{t}\right\}$ be an i.i.d. sequence with the following probability distribution:

(24) $$P\left(D_{t}=0\right)=p_{1},\;\;P\left(D_{t}=1\right)=p_{2},\;\;P\left(D_{t}=2\right)=1-p_{1}-p_{2},$$

where $p_{1}\geq 0$, $p_{2}\geq 0$ and $p_{1}+p_{2}\leq 1$. Then, the ZOBP autoregressive (ZOBPAR) model is defined as follows:

**Definition 18.**  *$\left\{B_{t}\right\}$ is said to follow the ZOBPAR model, if*

$$B_{t}=\left(2-D_{t}\right)D_{t}+\left(D_{t}-1\right)D_{t}B_{t}^{*}/2$$

with $B_{t}^{*}|{Ϝ}_{t-1}∼P^{*}\left(\lambda_{t},M\right)$ and

(25) $$\lambda_{t}=d+a\lambda_{t-1}+bB_{t-1}$$

where d>0, a≥0, b>0, and $D_{t}$ satisfying (12) is independent of $B_{t}^{*}$.

Compared with the model in vector form, the ZOBPAR model is concise both in terms of estimation and its own form. The follow-up Liu et al. [97] is also based on this and is an application-oriented study completed using Bayesian estimation methods.

## 5. Multivariate Integer-Valued Time Series

It can be seen that univariate INGARCH models have been well-studied in the literature, but progress in multivariate INGARCH models has lagged somewhat in comparison. It is encouraging to note that there have been some recent developments.

We start with a review of some studies based on further improvements of the bivariate Poisson (BP) INGARCH model. The definition of the BP-INGARCH model is given here.

**Definition 19.**  *Let $Y_{t}={\left(Y_{t,1},Y_{t,2}\right)}^{⊤}$. $\left\{Y_{t}\right\}$ is said to follow a BP-INGARCH(1,1) model if*

$$Y_{t}|{Ϝ}_{t-1}∼BP*(\lambda_{t}^{⊤},\phi ),\;\lambda_{t}={\left(\lambda_{t,1},\lambda_{t,2}\right)}^{⊤}=\omega +A\lambda_{t-1}+BY_{t-1},$$

where the definition of BP* is placed in Table 4, $\phi \geq 0,\omega ={\left(\omega_{1},\omega_{2}\right)}^{⊤}\in R_{+}^{2}$ and $A={\left\{\alpha_{ij}\right\}}_{i,j=1,2}$ and $B={\left\{\beta_{ij}\right\}}_{i,j=1,2}$ are 2×2 matrices with non-negative entries.

For the BP-INGARCH model, Lee et al. [25] showed the asymptotic normality of the conditional MLE and introduced the CUSUM test for parameter change based on the estimates and residuals. Additionally, Kim et al. [26] focused on a robust estimation method for BP-INGARCH models using the minimum density power divergence estimator.

The limitation of the BP-INGARCH model is that it can only handle positive cross-correlation between two components and is not competent for cross-correlations. A new BP-INGARCH model was proposed by Cui and Zhu [27] allowing for negative cross-correlation. Cui and Zhu [27] enabled an alternative definition of the BP distribution, just denoted as BP with definition given in Table 4, i.e., the product of Poisson marginals and a multiplicative factor δ that can promote positive, zero, or negative cross-correlation.

Further, a class of flexible BP-INGARCH(1,1) model was introduced by Cui et al. [98]. This class of models cover three distributions determined by different special multiplicative factors, making the portrayal of dependence more flexible.

**Definition 20.**  *A new class of BP-INGARCH(1,1) model with flexible multiplicative factor is proposed as follows:*

(26) $$Y_{t}|{Ϝ}_{t-1}∼GBP(\lambda_{t}^{⊤}),\lambda_{t}={\left(\lambda_{t,1},\lambda_{t,2}\right)}^{⊤}=\omega +A\lambda_{t-1}+BY_{t-1},$$

*where GBP(·) stands for one of three distributions, denoted as BPG($\lambda_{t}^{⊤},\rho$), BPF($\lambda_{t}^{⊤},\gamma$) and BPFGM($\lambda_{t}^{⊤},\sigma$) in Table 4.*

For correlated bivariate count time series data, it is worth mentioning that a new flexible bivariate conditional Poisson INGARCH model was recently proposed by Piancastelli et al. [99] to capture negative and positive cross-correlations as well. Although all of these models are flexible in terms of contemporaneous correlation, the explicit form of the correlation structure of Piancastelli et al. [99] is easier to assess. Piancastelli et al. [99] also provided a detailed comparison of these methods for bivariate count time series for reference.

The next review is no longer limited to bivariate INGARCH models, but multivariate INGARCH models. Multivariate count time series remains an area of research with a vast scope, both in terms of theoretical approaches and application-oriented research. Much of the existing methodological literature does not focus only on INGARCH models, but is more broadly applicable to various types of count time series models; see Fokianos [100] for some recent methodological developments including multivariate INGARCH models. Moreover, Fokianos et al. [101] introduced an overview of statistical analysis for some models for multivariate discrete-valued time series based on higher-order Markov chains, where several extensions are highlighted including non-stationarity, network autoregressions, conditional non-linear autoregressive models, robust estimation, random fields and spatio-temporal models. We only show the contribution made by the latest literature Lee et al. [102] because it was not included in Fokianos [100].

**Definition 21.**  *Let $Y_{t}={\left(Y_{t1},\cdots ,Y_{tm}\right)}^{⊤},t\geq 1$, be the time series of counts taking values in $N^{m}$, and*

$$p_{i}\left(y|\eta \right)=\exp\left\{\eta y-A_{i}\left(\eta \right)\right\}h_{i}\left(y\right),\;\;y\in N,$$

*which stands for the probability mass function of one-parameter exponential family, wherein η is the natural parameter, $A_{i}\left(\eta \right)$ and $h_{i}\left(y\right)$ are known functions, and both $A_{i}$ and $B_{i}=A_{i}^{{}^{\prime }}$ stands for the derivative of $A_{i}$, are strictly increasing. We then consider the following model.*

$$\begin{array}{cc} Y_{ti} & |{Ϝ}_{t-1}∼p_{i}\left(y|\eta_{ti}\right),\;i=1,\cdots ,m,\\ M_{t} & =E\left(Y_{t}|{Ϝ}_{t-1}\right)=f_{\theta }\left(M_{t-1},Y_{t-1}\right), \end{array}$$

*where $B_{i}\left(\eta_{ti}\right)=M_{ti}$, $f_{\theta }$ is a non-negative function defined on ${\left[0,\infty \right)}^{m}\times N^{m}$ depending on the parameter $\theta \in \Theta \subset R^{d}$ for some d=1,2,⋯, and $\eta_{t}={\left(\eta_{t1},\cdots ,\eta_{tm}\right)}^{⊤}:=B^{-1}\left(M_{t}\right):={\left(B_{1}^{-1}\left(M_{t1}\right),\cdots ,B_{m}^{-1}\left(M_{tm}\right)\right)}^{⊤}$.*

Unlike other authors who have devoted more effort to specifying the joint distribution of multivariate time series and the marginal distributions of their components, Lee et al. [102] argued that the conditional mean equation forms the bulk of the modeling and that the specification of the underlying joint distribution is not a major concern. We think this premise is a reasonable presupposition. It is well-known that the INGARCH-type model is built based on two types of assumptions, i.e., assumptions of conditional distribution and dynamic structure. Then it is intuitively reasonable that the contemporaneous dependence of the multivariate count time series can be reflected in the INGARCH model in two ways: the joint distribution and the multivariate dynamic structure. This presupposition reduces the complexity of modeling. Specially, although each component of $Y_{t}$ is modeled using a univariate INGARCH model in Definition 21, the dependence structure is imposed by the conditional mean process $M_{t}$.

Another emerging area is the development of INGARCH-type models applicable to time-varying network data, which can be considered as a special class of multivariate count time series. Therefore, a part of recent research has been devoted to establish INGARCH models and their methodologies applicable to time-varying discrete network data. Let $\left\{Y_{t}=\left(Y_{1t},\cdots ,Y_{Nt}\right)\right\}$ be a network with *N* nodes. The structure of the network is completely described by the adjacency matrix $A=\left(a_{ij}\right)\in R^{N\times N}$, where $a_{ii}=1$ for any i=1,⋯,N and $a_{ij}=1$ that means the presence of a directed edge from *i* to *j*, $a_{ij}=0$ otherwise. In general, any time-varying discrete network data whose relationship can be modeled by an adjacency matrix can be considered as a multivariate time series.

In a similar vein to the development of univariate integer-valued time series, the Poisson distribution and linearity assumptions continue to be used instinctively in order to establish a universal framework. Armillotta and Fokianos [28] considered the Poisson network autoregressive (PNAR) models for count data with a non-random adjacency matrix. We show here only the simplest linear form of order 1:

**Definition 22.**  *The PNAR(1) model is defined as:*

(27) $$\begin{array}{cc} Y_{i,t}|{Ϝ}_{t-1} & ∼Poisson(\lambda_{i,t}),\\ \lambda_{i,t} & =\beta_{0}+\beta_{1}n_{i}^{-1}\sum_{j=1}^{N}a_{ij}Y_{j,t-1}+\beta_{2}Y_{i,t-1}, \end{array}$$

*where $n_{i}=\sum_{i\neq j}a_{ij}$ is the out-degree, i.e., the total number of nodes, which i has an edge with.*

The PNAR(1) model reduces the inference complexity by incorporating network information into the dependence structure, where the response of each individual can be explained by its lagged values and the average effect of its neighbors in (27). Note that Equation (27) does not include information about the joint dependence structure of the PNAR(1) model. It is then convenient to rewrite (27) in vector form,

(28) $$Y_{t}=N_{t}\left(\lambda_{t}\right),\;\;\lambda_{t}={\vec{\beta }}_{0}+{\mathrm{GY}}_{t-1},$$

where ${\vec{\beta }}_{0}=\beta_{0}1_{N}\in R^{N}$, with $1={\left(1,1,\cdots ,1\right)}^{⊤}\in R^{N}$ and the matrix $G=\beta_{1}W+\beta_{2}I_{N}$, where $W=diag\{n_{1}^{-1},\cdots ,n_{N}^{-1}\}A$ is the row-normalized adjacency matrix, with $A=(a_{ij})$, so $w_{i}={\left(a_{ij}/n_{i},j=1,\cdots ,N\right)}^{⊤}\in R^{N}$ is the *i*-th row vector of the matrix W, satisfying ${∥W∥}_{\infty }=1$, and $I_{N}$ is the N×N identity matrix. $\left\{N_{t}\right\}$ is a sequence of independent *N*-variate copula–Poisson processes. The main methodological contribution of Armillotta and Fokianos [28] was the study of the asymptotic properties of such models by employing $L_{p}$-near epoch dependence and α-mixing, rather than based on the assumption that i.i.d. on which the development of all network time series models discussed so far has strongly relied. Further, Armillotta et al. [29] reviewed some of the work by Armillotta and Fokianos [28] and provided a unified framework for the statistical analysis of both continuous and integer-valued data with a known adjacency matrix. Armillotta and Fokianos [28] also specified a log-linear PNAR model for the count processes, and another recent work Armillotta and Fokianos [30] was closely related to this, where a quasi-score linearity test for continuous and count network autoregressive models was developed.

It can be seen in (27) and (28) that the PNAR model assumes that all individuals are homogeneous and they share a common autoregressive coefficient. This is a somewhat detached assumption from reality. Therefore, Tao et al. [31] proposed the grouped PNAR, which divides individuals into different groups and describes heterogeneous node behavior with group-specific parameters. Compared to the original PNAR model, the constraints are relaxed while competently portraying the heterogeneity. Specially, all individuals could be classified into *K* groups in the setting of Tao et al. [31], and each group was characterized by a specific set of positive parameters $\theta_{k}={\left(\omega_{k},\alpha_{k},\rho_{k},\beta_{k}\right)}^{\prime }\in R^{4}$, for 1≤k≤K.

**Definition 23.**  *A grouped PNAR model can be constructed as*

(29) $$\begin{array}{cc} Y_{i,t}|{Ϝ}_{t-1} & ∼Poisson(\lambda_{i,t}), \end{array}$$

(30) $$\begin{array}{cc} \lambda_{i,t} & =\sum_{k=1}^{K}z_{ik}\left(\omega_{k}+\alpha_{k}Y_{i,t-1}+\rho_{k}d_{i}^{-1}\sum_{j\neq i}a_{ij}Y_{j,t-1}+\beta_{k}\lambda_{i,t-1}\right), \end{array}$$

*for each i=1,⋯,N, and t≥1. Following the PNAR model, the parameters $\omega_{k},\alpha_{k},\rho_{k},\beta_{k}$ represent the group-specific baseline effect, regression coefficient on past observations, network effect, and regression coefficient on past intensity processes, respectively. Note that we assume the adjacency matrix A is asymmetric, which covers the special case of symmetric networks. To distinguish between groups, latent variable $z_{ik}\in \left\{0,1\right\}$ was defined for each object i, where $z_{ik}=1$ if object i is from the k-th group, and $z_{ik}=0$ otherwise. Assume $\left\{{\left(z_{i1},\cdots ,z_{iK}\right)}^{\prime },1\leq i\leq N\right\}$ is a sequence of i.i.d. multinomial random vectors with number of events n=1 and probability $\gamma ={\left(\gamma_{1},\cdots ,\gamma_{K}\right)}^{\prime }$. Here, $\gamma_{k}$ represents the group proportion satisfying $\gamma_{k}\geq 0$ and $\sum_{k=1}^{K}\gamma_{k}=1$.*

Tao et al. [31] explored the accuracy of model estimation and prediction when the group labels were unknown and the number of group *K* is mis-specified, respectively. There is already a body of mature research on network data, but it is still a relatively emerging topic in the INGARCH field. Therefore, many further attempts to consider time-varying networks from the perspective of count time series are worthwhile, such as optimization of conditional distributions, nonlinear dynamic structures, and related hypothesis testing, or time-varying network data with upper bounds on the number of edges.

In addition to modeling and methodology, INGARCH is popular for application-oriented analysis of time-varying networks. For example, Agosto and Ahelegbey [103] used a financial network model to study the contagion effects between business sectors based on discrete data, and tested the conditional means (and volatilities) of default counts across economic sectors estimated by Poisson INGARCH and their dependence in shocks. Through an empirical analysis of corporate defaults in Italy over the period 1996–2018, a high degree of intersectoral vulnerability was concluded by Agosto and Ahelegbey [103], in particular at the onset of the global financial crisis in 2008 and in subsequent years. Such a wide range of application prospects is accompanied by a desire for theoretical development.

## 6. Discussion and Conclusions

**Figure 3:**
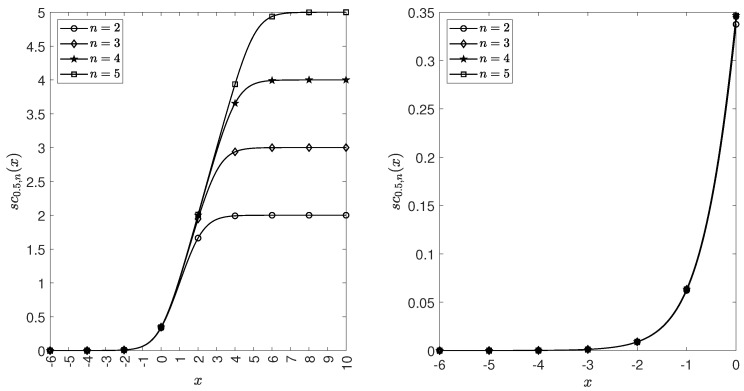
Plots of soft clipping functions $sc_{\frac{c}{n}}\left(x\right),\;x\in \left[-6,10\right]$ (**left**) and $sc_{\frac{c}{n}}\left(x\right),\;x\in \left[-6,0\right]$ (**right**) with c=0.5 and n=2,3,4,5.

**Figure 4:**
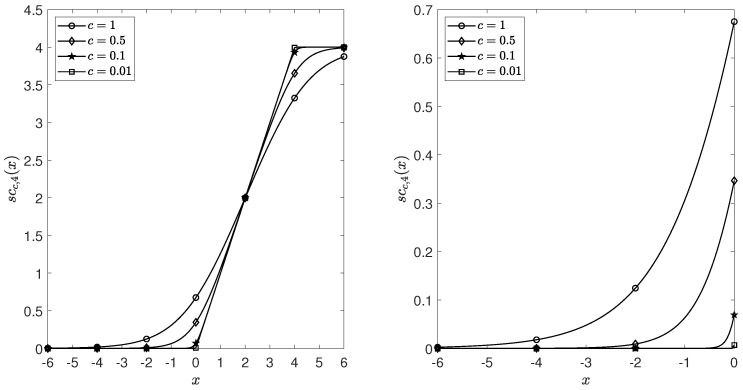
Plots of soft clipping functions $sc_{\frac{c}{n}}\left(x\right),\;x\in \left[-6,6\right]$ (**left**) and $sc_{\frac{c}{n}}\left(x\right),\;x\in \left[-6,0\right]$ (**right**) with n=4 and c=1,0.5,0.1,0.01.

The purpose of this section is to present some potentially useful research topics based on the methodology and applications reviewed in the previous sections.

(1). First, we focus on the softplus function $sp_{c}\left(\cdot \right)$ and soft-clipping function $sc_{\frac{c}{n}}\left(\cdot \right)$, which contribute to the modeling of unbounded counts and bounded counts allowing for negative auto-correlation, respectively. For the sake of clarity, $sc_{\frac{c}{n}}\left(\cdot \right)$ is used next as an example. As already mentioned, the advantage of $sc_{\frac{c}{n}}\left(\cdot \right)$ is that the support set is R and is nearly linear on (0,n], which allows the parameters in the dynamic structure of the BINGARCH model not to be restricted to positive numbers. This is certainly an excellent innovation, and one that seems worth exploring further. The images of $sc_{\frac{c}{n}}\left(x\right)$ corresponding to different parameters *c* or *n* are reported in Figure 3 and Figure 4. It is obvious that the slope of $sc_{\frac{c}{n}}\left(x\right)$ is small when x<0 and tends to zero as *x* decreases. Moreover, the parameter *c* has a small moderating effect on this tendency, while *n* even has almost no effect. This insensitivity to negative values may lead to concerns that the corresponding INGARCH models do not fairly model positively and negatively correlated data.

The reasons for this confusion can be summarized as follows. Although negative parameters are allowed to appear in the dynamic structure of the conditional expectation, such leniency seems to be somewhat offset by the fact that $P_{t}$ (we use $Bin(n,p_{t})$ as an example here) that were obtained from $sc_{\frac{c}{n}}\left(x\right)$ are concentrated around 0 when x<0, while $sc_{\frac{c}{n}}\left(x\right)$ is approximately linear when x>0. The $sc_{\frac{c}{n}}\left(x\right)$ does allow for the existence of negative correlation, but the extent of the negative correlation is to be explored further. However, to put it another way, it seems to us that $sc_{\frac{c}{n}}\left(x\right)$ provides a new perspective of modeling zero-inflated counts. In contrast to the previous idea of embodying the zero-inflated feature in a conditional distribution, it seems possible to assign this task to a dynamic structure through $sc_{\frac{c}{n}}\left(x\right)$.

(2). There is still a large demand for innovations dedicated to bounded count time series. We personally think that there are two feasible directions for exploration: one is the innovation of conditional distribution, and the other is to continue to deepen the research based on vector forms. From an application point of view, it is worth exploring to truncate existing distributions in a reasonable way, or to find distributions that themselves take values in bounded sets of integers. For the study of vector forms, where the theory is relatively well-developed, it is imperative to ease the estimation pressure.

(3). The emergence of new methodologies or research topics in other fields or in the broader field can stimulate the development of the INGARCH-type models. For example, Pedersen and Rahbek [104] presented the theory for testing for reduction of GARCHX type models with an exogenous covariate to standard GARCH type models. Many INGARCH-type models, including some of the recent literature mentioned earlier, are also focusing on covariates. Thus, the tests and methodologies proposed by Pedersen and Rahbek [104] can actually inspire existing INGARCH models to achieve tests of the reasonableness of introducing covariates. Similarly, refer also to Aknouche and Francq [105] and Debaly and Truquet [106].

## Figures and Tables

**Table 1:** Summary of basic properties of distributions related to INGARCH.

Distribution	Definition
BNB (Gorgi [7])	The probability mass function (PMF) of BNB is $$P\left(X=x\right)=\frac{\Gamma \left(x+r\right)}{\Gamma \left(x+1\right)\Gamma \left(r\right)}\frac{B(\alpha +r,\beta +x)}{B(\alpha ,\beta )},$$ where Γ(·) and B(·,·) denote the gamma function and beta function, respectively. And $E\left(X\right)=\frac{\beta r}{\alpha -1}$.
GCOMP (Qian and Zhu [8])	The PMF of GCOMP is is $$P\left(X=x\right)=\frac{\Gamma {\left(v+x\right)}^{r}\zeta^{x}}{x!C(r,\nu ,\zeta )},$$ where the normalizing constant $C\left(r,\nu ,\zeta \right)=\sum_{k=0}^{\infty }\frac{\Gamma {\left(\nu +k\right)}^{r}\zeta^{k}}{k!}$, for r<1, v>0 and ζ>0 or r=1,v>0 and 0<ζ<1. $E\left(X\right)=\frac{C(r,\nu +1,\zeta )\zeta }{C(r,\nu ,\zeta )}$ and $Var\left(X\right)=\frac{C\left(r,\nu +2,\zeta \right)\zeta^{2}}{C(r,\nu ,\zeta )}+\frac{C(r,\nu +1,\zeta )\zeta }{C(r,\nu ,\zeta )}-\frac{C^{2}\left(r,\nu +1,\zeta \right)\zeta^{2}}{C^{2}\left(r,\nu ,\zeta \right)}$
MP (Silva and Souza [9])	Let *Z* be a continuous positive random variable belonging to the exponential family with density function given by $$f\left(z\right)=\exp\{\phi \left[z\zeta_{0}-b\left(\zeta_{0}\right)\right]+c\left(z;\phi \right)\},$$ where b(·) is a continuous three-times differentiable function and $\zeta_{0}$ is such that $b^{\prime }\left(\zeta_{0}\right)=1$ and c(·;·) is a function that maps $R^{+}\times R^{+}$ into *R*. Thus, $E\left(Z\right)=b^{\prime }\left(\zeta_{0}\right)=1$ and $Var\left(Z\right)=\phi^{-1}b^{\prime \prime }\left(\zeta_{0}\right)$. We denote this as Z∼EF(ϕ). Let X|Z=z∼Poisson(μz), with μ>0 and Z∼EF(ϕ). Then, *X* belongs to the class of mixed Poisson distributions. If *Z* follows a gamma distribution with mean 1 and dispersion parameter ϕ, we find that *X* follows a negative binomial distribution with parameters μ and ϕ. Its probability function is given by $$p\left(x;\mu ,\phi \right)=\frac{\Gamma (x+\phi )}{x!\Gamma (\phi )}{\left(\frac{\mu }{\mu +\phi }\right)}^{x}{\left(\frac{\phi }{\mu +\phi }\right)}^{\phi }.$$

**Table 2:** Summary of basic properties of distributions related to Z-valued INGARCH.

Distribution	Definition
Skellam (Alomani et al. [91])	The PMF of Skellam is $$P_{S}\left(Z=z\right)=exp\left\{-\left(\lambda_{1}+\lambda_{2}\right)\right\}{\left(\frac{\lambda_{1}}{\lambda_{2}}\right)}^{z/2}I_{|z|}\left(2\sqrt{\lambda_{1}\lambda_{2}}\right),$$ where $I_{r}\left(x\right)$ is the modified Bessel function of order *r* and is defined by $I_{r}\left(x\right)={\left(\frac{x}{2}\right)}^{r}\sum_{k=0}^{\infty }\frac{{\left(x^{2}/4\right)}^{k}}{k!\Gamma (r+k+1)}$. And $E\left(Z\right)=\lambda_{1}-\lambda_{2}$ and $Var\left(Z\right)=\lambda_{1}+\lambda_{2}.$
MS (Cui et al. [92])	The PMF of MS is $$P_{MS}\left(Z=z\right)=f_{MS}\left(z|\lambda_{1},\lambda_{2}\right)=\left\{\begin{array}{c} P_{S}\left(Z=z\right),\;\;z\in Z/\left\{0,\pm 1\right\}\\ P_{S}\left(Z=z\right)-\frac{1}{2}\gamma \Delta ,\;\;z=-1\;or\;1\\ P_{s}\left(Z=z\right)+\gamma \Delta ,\;\;z=0 \end{array}\right.$$ where $▵=P_{s}\left(Z=0\right)-\min\left\{P_{s}\left(Z=-1\right),P_{s}\left(Z=1\right)\right\}>0$ and $P_{s}\left(Z=q\right)=f_{s}\left(q|\lambda_{1},\lambda_{2}\right)$ for q∈Z. $E\left(Z\right)=\lambda_{1}-\lambda_{2}$ and $Var\left(Z\right)=\lambda_{1}+\lambda_{2}-\gamma \Delta .$
SGe (Xu and Zhu [20])	The PMF of SGe is $$P\left(X=k\right)=p{\left(1-p\right)}^{k-1},k=1,2,3,\cdots$$ E(Z)=1/p and $Var\left(Z\right)=\left(1-p\right)/p^{2}$

**Table 3:** Summary of basic properties of distributions related to BINGARCH.

Distribution	Definition
BB (Chen et al. [23])	The PMF of BB is P(Z=z)=nzB(z+a,n−z+a)B(a,b) with B(a,b)=Γ(a)Γ(b)Γ(a+b). The beta-binomial distribution approximately reduces to the usual binomial distribution when a→∞ or b→∞. $E\left(Z\right)=\frac{na}{a+b}$ and $Var\left(Z\right)=\frac{nab}{{\left(a+b\right)}^{2}}\left(1+\frac{n-1}{a+b+1}\right)$
DB (Chen [95])	The PMF of DB is $$P\left(X=x|\alpha ,\beta ,n\right)=\frac{1}{Z(\alpha ,\beta )}f\left(\frac{x-n_{bot}+1}{n_{top}-n_{bot}+2}\right),$$ where $$f\left(x\right)=\frac{1}{B(\alpha ,\beta )}x^{\alpha -1}{\left(1-x\right)}^{\beta -1},Z\left(\alpha ,\beta \right)=\sum_{x=n_{bot}}^{n_{top}}f\left(\frac{x-n_{bot}+1}{n_{top}-n_{bot}+2}\right),$$ $n_{top}\in N$ is the predetermined upper limit of the range and $n_{bot}=0$ or 1 is the predetermined lower limit of the range (*Z* taking values in $\left\{n_{bot},n_{bot}+1,n_{bot}+2,\ldots ..,n_{top}\right\}$)

**Table 4:** Summary of basic properties of distributions related to MINGARCH.

Distribution	Definition
BP* (Lee et al. [25])	Consider random variables $X_{k}$, k=1,2,3, which follow independent Poisson distributions with parameters $\lambda_{1}-\phi$, $\lambda_{2}-\phi$, ϕ, respectively, and then the random variables $Y_{1}=X_{1}+X_{3}$ and $Y_{2}=X_{2}+X_{3}$ jointly follow a bivariate Poisson distribution BP*$\left(\lambda_{1},\lambda_{2},\phi \right)$ with probability mass function: P(Y1=y1,Y2=y2)=e−(λ1+λ2−ϕ)(λ1−ϕ)y1(λ2−ϕ)y2y1!y2!×∑k=0min(y1,y2)y1ky2kk!ϕ(λ1−ϕ)(λ2−ϕ)k, with $E\left(Y_{1}\right)=Var\left(Y_{1}\right)=\lambda_{1}$, $E\left(Y_{2}\right)=Var\left(Y_{2}\right)=\lambda_{2}$ and $Cov(Y_{1},Y_{2})=\phi$.
BP (Cui and Zhu [27])	A bivariate Poisson distribution is defined as a product of Poisson marginals with a multiplicative factor δ, whose PMF is given by $$P\left(Y_{1}=y_{1},Y_{2}=y_{2}\right)=\frac{\lambda_{1}^{y_{1}}\lambda_{2}^{y_{2}}}{y_{1}!y_{2}!}e^{-(\lambda_{1}+\lambda_{2})}\left[1+\delta \left(e^{-y_{1}}-e^{-c\lambda_{1}}\right)\left(e^{-y_{2}}-e^{-c\lambda_{2}}\right)\right]$$ where $c=1-e^{-1}$, with $E\left(Y_{1}\right)=Var\left(Y_{1}\right)=\lambda_{1}$, $E\left(Y_{2}\right)=Var\left(Y_{2}\right)=\lambda_{2}$ and $Cov\left(Y_{1},Y_{2}\right)=\delta c^{2}\lambda_{1}\lambda_{2}e^{-c(\lambda_{1}+\lambda_{2})}$.
BPG (Cui et al. [98])	The PMF of BPG is $$P\left(Y_{1}=y_{1},Y_{2}=y_{2}\right)=\frac{1}{Z(\lambda_{1},\lambda_{2},\theta )}\frac{\lambda_{1}^{y_{1}}\lambda_{2}^{y_{2}}}{y_{1}!y_{2}!}e^{-(\lambda_{1}+\lambda_{2})}c_{\rho }\left(F_{1}\left(y_{1}\right),F_{2}\left(y_{2}\right)\right),$$ where $Z(\lambda_{1},\lambda_{2},\theta )$ is the normalizing factor, $$c_{\rho }\left(\mu_{1},\mu_{2}\right)=\frac{1}{\sqrt{1-\rho^{2}}}\exp\left(-\frac{\rho^{2}\left(q_{1}^{2}+q_{2}^{2}\right)-2\rho q_{1}q_{2}}{2(1-\rho^{2})}\right)$$ $q_{i}=\phi^{-1}\left(u_{i}\right)$, $\phi^{-1}$ is the inverse of the standard univariate normal distribution, $\mu_{i}\in \left[0,1\right]$ for i=1,2, ρ∈(−1,1), γ∈(−∞,∞)/{0} and σ∈(−1,1) are regarded as dependency parameters for BP. Let $$c_{\gamma }\left(\mu_{1},\mu_{2}\right)=\frac{-\gamma \left(e^{-\gamma -1}\right)e^{-(\mu_{1}+\mu_{2})\gamma }}{{\left[\left(e^{-\gamma }-1\right)+\left(e^{-\mu_{1}\gamma }-1\right)\left(e^{-\mu_{2}\gamma }-1\right)\right]}^{2}}$$ and $$c_{\sigma }\left(\mu_{1},\mu_{2}\right)=1+\sigma \left(1-2\mu_{1}\right)\left(1-2\mu_{2}\right),$$ and then replacing $c_{\rho }\left(F_{1}\left(y_{1}\right),F_{2}\left(y_{2}\right)\right)$ with $c_{\gamma }\left(F_{1}\left(y_{1}\right),F_{2}\left(y_{2}\right)\right)$ and $c_{\sigma }\left(F_{1}\left(y_{1}\right),F_{2}\left(y_{2}\right)\right)$ yields the PMF of BPF and that of BPFGM, respectively.

## Data Availability

No new data were created in this review.
